# Financial Fraud Detection and Prediction in Listed Companies Using SMOTE and Machine Learning Algorithms

**DOI:** 10.3390/e24081157

**Published:** 2022-08-19

**Authors:** Zhihong Zhao, Tongyuan Bai

**Affiliations:** 1 School of Applied Science and Civil Engineering, Beijing Institute of Technology, Zhuhai 519085, China; 2 Faculty of Natural, Mathematical and Engineering Sciences, King’s College, London WC2R 2LS, UK

**Keywords:** financial fraud, feature selection, classification algorithms, grid search, voting

## Abstract

This paper proposes a new method that can identify and predict financial fraud among listed companies based on machine learning. We collected 18,060 transactions and 363 indicators of finance, including 362 financial variables and a class variable. Then, we eliminated 9 indicators which were not related to financial fraud and processed the missing values. After that, we extracted 13 indicators from 353 indicators which have a big impact on financial fraud based on multiple feature selection models and the frequency of occurrence of features in all algorithms. Then, we established five single classification models and three ensemble models for the prediction of financial fraud records of listed companies, including LR, RF, XGBOOST, SVM, and DT and ensemble models with a voting classifier. Finally, we chose the optimal single model from five machine learning algorithms and the best ensemble model among all hybrid models. In choosing the model parameter, optimal parameters were selected by using the grid search method and comparing several evaluation metrics of models. The results determined the accuracy of the optimal single model to be in a range from 97% to 99%, and that of the ensemble models as higher than 99%. This shows that the optimal ensemble model performs well and can efficiently predict and detect fraudulent activity of companies. Thus, a hybrid model which combines a logistic regression model with an XGBOOST model is the best among all models. In the future, it will not only be able to predict fraudulent behavior in company management but also reduce the burden of doing so.

## 1. Introduction

As the economy grows rapidly, the number of listed companies in the world is increasing year by year. At the same time, more and more companies have financial problems than before. It is not only highly detrimental to the image of companies and their management but also has a negative impact on investors and shareholders. Financial fraud [1] refers to intentional manipulation, falsification, or modification of documents by the accountant or senior manager of the company. Sometimes, they even ignore or delete important information from financial statements, which can have a significant impact on the management of the company.

There have been many previous studies on financial fraud. At the end of the last century, Bologne G. Jack. and Wells, Joseph T. (1993) [2] suggested the GONE theory which includes four factors, such as greed, opportunity, need, and exposure. Greed means that the person are interested in money or other attractions instead of protecting data. Opportunities and needs refer to the promotion and the income or other welfare from working. Exposure means the consequences of wrong actions in financial data. In 1987, the National Commission on Fraudulent Financial Reporting (NCFFR) in the United States [3] also pointed out that accounting fraud can easily lead to erroneous decisions. At the same time, fake data can cause damage to investor ownership. In a 2002 review, Renhua Li [4] found that some fraudulent behavior has a significant negative effect on revenues. Moreover, Handoko [5] found that financial difficulties, such as income stability and management of liquidity also have a significant impact on financial fraud.

In China, Luckin Coffee [6] increased its sales revenue and recorded the false price of a cup of coffee. In February 2020, Muddy Waters published a report about the behavior of financial fraud and the company admitted financial fraud and moved out of the stock market on May 19. Wanfushengke [7] entered the main board market and managers falsified the sales data and were under investigation by the competent authorities in 2012. Finally, it was punished by the government and never allowed into the stock market. Zhangzi Island [8] used natural factors to conceal property losses caused by human factors. At the same time, investigators lacked relevant information and data to identify fraudulent acts indicated by false data.

In this article, we set up 363 indicators, including one interest variable and 362 other variables. The dependent variable is a class variable. When the value is 0, it means that the company is legitimate. When the value is 1, it indicates fraudulent activity of the company. In addition, these independent variables are related to the state of finance of the company. In that case, we must do our best to select important independent indicators of financial state that can detect financial fraud immediately and rapidly. In this paper, we extracted the most relevant indicators and built a classification model by using machine learning (ML) algorithms that can effectively identify and predict whether or not the company has committed financial data fraud, such as Logistic Regression (LR), Support Vector Machine (SVM), Random Forest (RF), and Extreme Gradient Boosting (XGBOOST). In addition, we also proposed a voting classifier that can use two classification models at the same time. The principal contribution of this research may be summarised as follows:

- We have comprehensively investigated the issues of fraud detection in listed companies related to imbalanced data classification in machine learning.
- The number of financial fraud records is 18,060 and the number of indicators is 353. This means that a large amount of data is the basis of our research; thus increasing the probability of accurate prediction.
- We used the Synthetic Minority Oversampling Technique (SMOTE) to address imbalance in the data set.
- We proposed the new framework when we chose reasonable indicators which have a big impact on the type of companies, such as Logistic Regression (LR), Random Forest (RF), Gradient Boosting Decision Tree (GBDT), Decision Tree (DT), and Extreme Gradient Boosting (XGBOOST).
- We carried out grid search that can choose the best parameter when constructing machine learning models, including Logistic Regression (LR), Support Vector Machine (SVM), Random Forest (RF), and Extreme Gradient Boosting (XGBOOST).
- We implemented voting classifier method with several ML methods to get more reasonable and scientific results compared with single classification models of reviews.

The rest of this paper is structured as follows. Related work is reviewed in the Section 2. Then, we plan to introduce the data set and describe the framework of research about how to select features and construct single and multiple classifiers and evaluate the performance of them in the Section 3. As for the Section 4, we will discuss and analyse these results. Finally, we will draw up a conclusion in the final part.

## 2. Related Work

Currently, a variety of factors affect the detection of financial fraud. It is necessary for us to build feature selection models and classification models based on a number of samples and numerous indicators, which can increase the accuracy of prediction compared with previous work and provide meaningful suggestions for investors and authorities.

Imbalanced data sets have been a significant challenge in recent years. Mohammed et al. [9] built several machine learning models without choosing their parameters. The results showed that Random Forest, Balanced Bagging Ensemble, and Gaussian Naïve Bayes have a good performance. However, it is difficult to maintain similar results in massive data sets. Kaur et al. [10] proposed a review for the solutions of imbalanced data sets and the pros and cons of machine learning, including neural networks, k-nearest neighbour(KNN), and so on. SMOTE is a better method compared with the under-sampling and hybrid sampling methods. Ganganwar [11] found that current research in imbalanced data sets are focusing on hybrid algorithms, such as bagging models and ensemble models.

In terms of feature selection models, Neumann et al. [12] suggested new approaches to the feature selection, including linear and Support Vector Machine classifiers. At the same time, they applied the different convex function, a general framework for non-convex continuous optimization. Tang et al. [13] studied different methods of feature selection for different types of features, such as character or numeric data. In terms of the method of feature selection, researchers can use filter models, wrapper models and embedded models. For streaming functions, they used the graft algorithm, alpha investing algorithms and selection of online streaming functions. Guyon et al. [14] proposed evaluation criteria for feature selection prior to building machine learning models, such as the importance of indicators and how to establish correlation criteria. Then, these researchers pay attention to the type of variable when selecting features. Wrappers are used to choose features according to the performance of the classifier. Filters worked as a pre-processing step and in embedded ways select variables during the training process. Kohavi et al. [15] studied the filter method and compared it with the pros and cons of a wrapper method. The result showed that the latter can improve accuracy in decision trees and naive-Bayes. Omar et al. [16] studied classification, whether using feature selection or not. The result showed a higher accuracy and reduced classifier workload by selection of the relevant variable. Coelho et al. [17] proposed a new estimator based on mutual information that can process both continuous and discrete variables simultaneously. It is always used for pattern recognition and feature selection.

In terms of classification models, Bell et al. [18] studied companies in the field of finance and technology and used logistic regression models for analysis. It was proven that income fell as the risk of the company’s financial data fraud increases substantially. If the rate of increase is too high, there is a high risk of fraud. Spathis et al. [19] studied 10 indicators of funding of 76 enterprises and used a multi-criteria analysis method to evaluate the performance and a multivariate statistical method for analysis. The results showed that indicator selection plays an important role in detecting the class of companies. Kirkos E et al. [20] selected 10 factors in 76 Greek manufacturing companies and carried out the Decision Tree, Neural Network Model, and Bayesian Network model to conduct experiments. The results showed that the accuracy of Bayesian network model at 90.3%. It means that the Bayesian network model can predict more accurately than other models whether the company has fraudulent financial data or not. CJ Skousen et al. [21] selected companies which were punished by the US Securities and Exchange Commission from 1992 to 2001 as the research object and built the logistic regression model based on the fraud triangle theory. It showed that there was a relationship between the demand for cash and financial fraud. Ravisankar et al. [22] used data mining techniques such as Multilayer Feed Forward Neural Network (MLFF), Support Vector Machines (SVM), and so on to identify financial statements. Glancy et al. [23] proposed a computational fraud detection model (CFDM) for detecting fraud in financial reporting.

In summary, the researchers compared different algorithms to address the issue of imbalanced data sets and detect fraud. The studies of the researchers mentioned cannot set up the best parameters of machine learning methods which play an important in performance of models. At the same time, when most research focused on the performance metrics, they only used the accuracy as the main indicator instead of many different performance metrics. Therefore, we need to build various classification models based on large samples and many indicators in the data. In addition, we evaluated these models with performance metrics. Neural network is quite diffcult to explain the meaning of each indicator and the running time is longer than machine learning models. We proposed Voting which used two different algorithms. This paper selected the model with the best performance for identifying and predicting financial fraud of listed companies which is beneficial to make up for this shortcoming.

## 3. Research Methology

### 3.1. Framework

**Figure 1:**
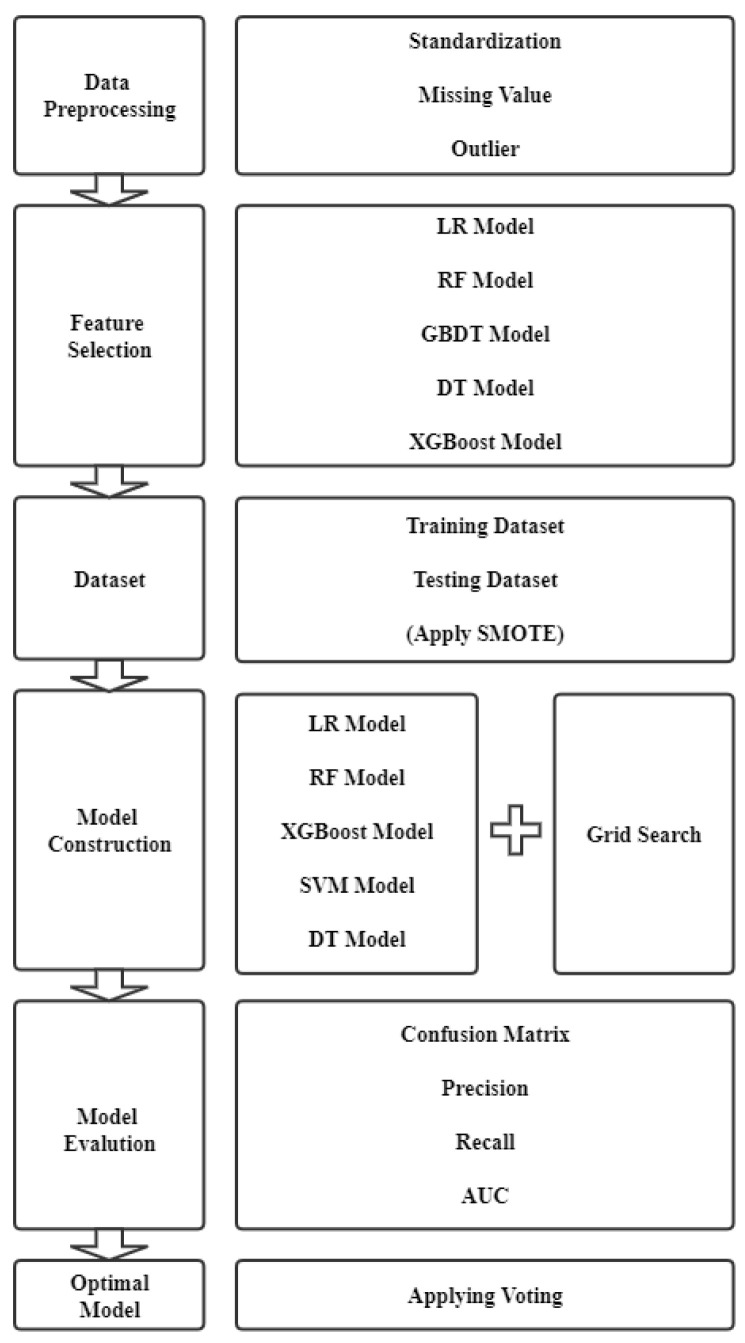
The overall framework of the proposed intelligent approach for fraud detection.

This paper divides listed companies into two types, such as fraudulent company and common company. We used machine learning methods to select the financial indicators of listed companies and build machine learning prediction models and evaluate results according to performance metrics. The process for this paper is divided into five phases. Firstly, we processed data, such as missing values and outliers. Then, we used Logistic Regression (LR), Random Forest (RF), Gradient Boosting Decision Tree (GBDT), Decision Tree (DT), and Extreme Gradient Boosting (XGBOOST) models to select the top 20% of features after preprocessing and building models. In addition, we counted the frequency of these indicators when we extracted. When the frequency is higher than 4 times, the indicator will be chosen. After selecting, we applied the Synthetic Minority Over-sampling Technique (SMOTE) method to ensure a balance of the class type of samples between training data set and testing data set. In the fourth step, we used the Logistic Regression (LR), Random Forest (RF), Extreme Gradient Boosting (XGBOOST), Support Vector Machine (SVM), and Decision Tree (DT) to predict whether the company has financial fraud problems or not. Then, we used grid search to choose the best parameter of each model and evaluate these models with four performance metrics, such as accuracy, recall, precision and area under curve (AUC). Finally, we chose the best machine learning models from four models and combined them with majority voting which have better performance. The overall framework of the proposed intelligent approach for fraud detection is shown in Figure 1.

### 3.2. Dataset and Data Pre-Processing

The data set was downloaded from the contest website, the 9th TipDM Cup Data Mining Challenge. This data set was collected financial information of stock and bond of listed companies from the stock market in Shanghai and Shenzhen and the Hong Kong stock market. The data set contains 18,060 samples. All indicators are grouped into three areas, including cash flow, operating capacity, and profitability. After calculating, 98.99% of companies are legitimate and 1.01% of companies are fraudulent. Additionally, it contains 363 indicators ($x_{1},x_{2},x_{3},\ldots ,x_{363}$) about finance and management of business. There is a class indicator called FLAG. When the value is 0 it indicates a legitimate company and a value of 1 indicates a fraudulent company.

In the real world, it is difficult for us to collect all indicators of all companies at the same time because it depends on the accountant, the year of establishment, and so on. In other words, there are many factors that have a big impact on the collection of data. In that case, we need to process the data, including the missing value. In addition, there is no direct link between some indicators and the type of company by analysing the means of all indicators. Therefore, we excluded these indicators, such as ticker_symbol, act_pubtime, publish_date, end_date_rep, report_type, fiscal_period, merged_flag, accouting_standards, currency_cd. After processing, there are still 353 features in the data set. If there are many missing values of each indicator, it is not conducive to the analysis of the importance of the features in the model. We proposed a new way in previous work. When the percent of the missing value of feature is greater than 70%, we decided to delete it. When that of the rest of feature is in the range from 40% to 70%, we will use 0 to fill the missing values. Finally, when the proportion of the missing value is less than 40%, we use the mean filling method to fill the missing values.

After processing using these methods, we extracted 240 effective indicators among all variables. At the same time, there are no missing values among all features. Then, we calculated the percentage complete for each feature and the percentage of missing at 0%. In other words, the percentage of complete accounts for 100%. This depicted that the percentage of complete among all the retained features is 100%. In other words, these indicators are vaild.

In addition, we found that the unit of each indicator varies from indicator to indicator, so we used standardisation to scale the data to between 0 and 1, which can decrease the probability of the accuracy of models due to different scales. We defined that ${\bar{x}}_{j}$ is the *j*th indicator, $s_{j}$ is the standard deviation of the *j*th indicator, $x_{ij}^{*}$ is the value of the *i*th sample in the *j*th indicator and z-score equation as shown in (1).

(1) $$x_{ij}^{*}=\frac{x_{ij}-{\bar{x}}_{j}}{s_{j}}\left(i=1,2,\ldots ,18,060,\;j=1,2,\ldots ,240\right)$$

### 3.3. Feature Selection

Selecting significant and important features has a positive effect on classifying companies as having committed financial fraud or not. Tang [13] studied the field of feature selection and proposed three types of feature selection for classification, such as filter models, wrapper models, and embedded models.

**Figure 2:**
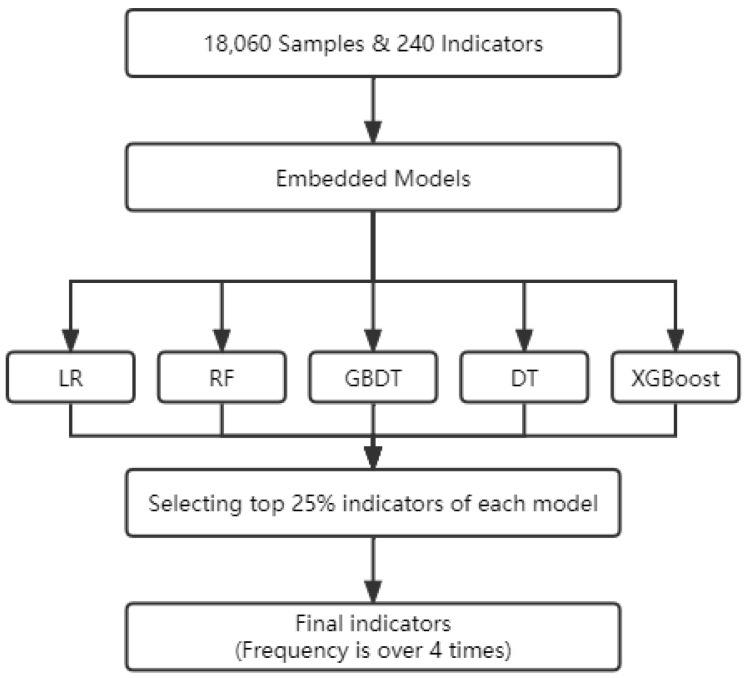
The overall framework of feature selection.

Finally, we decided to use embedded models by comparing strengths and limitations of three models, such as Logistic Regression (LR), Random Forest (RF), Gradient Boosting Decision Tree (GBDT), Decision Tree (DT), and Extreme Gradient Boosting (XGBOOST). Firstly, we inputed 240 indicators into each models and selected top 25% indicators at about 60 variables. Then, we selected the most frequent indicators by the frequency of those that occurred more than 4 times. This means that these are the most significant and relevant. The overall framework of feature selection is shown in Figure 2.

According to Figure 2, we used five different methods that can select features from data set. Then, we explained the details of standards of selection. In Logistic Regression model and Extreme Gradient Boosting model, we trained model and sorted them by the weight of independent variables. In addition, we sorted them by the gini index in Random Forest, Gradient Boosting Decision Tree, and Decision Tree. The equation is shown in (2)

(2) $$Gini\left(D\right)=1-\sum_{k=1}^{K}p_{k}^{2}$$

In Equation (2), *k* denotes that the number of type and $p_{i}$ means that the percentage of the *i*th type. When the gini is higher, it means that the system or model is not steady. At the same time, these three models are tree models and it is clear that the gini can select features more effectively. In addition, we also did some experiments on selecting by information entropy instead of gini. There are two disadvantages about information entropy. Firstly, the speed of calculation is lower. Thus, gini is more suitable when we need to build tree models.

### 3.4. Sampling Method

It is quite common to find imbalances of samples in the field of finance, industry, and business. In general, we defined the data set as *S*, the major samples in one class are $S_{maj}$, others are $S_{min}$. This means that the proportion of one class is higher than that of another. In such cases, we used Synthetic Minority Oversampling Technique (SMOTE), which can address this problem effectively. SMOTE is one of the oversampling techiques that was introduced by Chawla et al. [24]. The SMOTE method [25,26] identifies the feature vector $x_{i}$ and identifies the k-nearest neighbors $x_{knn}$. Then, we calculated the difference between the feature vector and neighbors. In the next step, we multiplied the difference by a random number between 0 and 1 and adds the output number to feature vector to identify a new point on the line segment. The equation is shown in (3) [25].

(3) $$x_{new}=x_{i}+\left(x_{knn}-x_{i}\right)\times t$$

### 3.5. Machine Learning Models

The aim is to predict the type of company by using machine learning models. Thus, we will introduce the details of several models, such as LR, RF, XGBOOST, SVM, and DT.

Logistic classification [27] can predict whether a company has financial fraud or not by inputting many variables. In addition, it is not only can not be affected by slight multicollinearity but also it can analyse large data while using fewer resources. In this model, we set the best parameter by cross entropy, which is very popular in classification models, such as Logistics Regression model and Neural Networks. The equation is shown in (4).

(4) $$C_{CE}\left(p,q\right)=-\sum_{x}p\left(x\right)\log\left(q\left(x\right)\right)$$

Equation (4) shows that *p* is the predicting value and *q* is the true value. When *p* is getting close to *q*, the loss function is the lowest.

The random forest model [28] is an ensemble learning model that can analyze data that have numerous features. In general, the algorithm selects the random subset of features for training once. At the same time, it can save time and is easily implemented compared with others. In addition, the algorithm also introduces randomness, which can effectively avoid the over-fitting phenomenon.

The extreme gradient boosting model [29] was proposed by Chen and Guestrin, which is a kind of gradient boosting algorithm. It breaks the computational limitations of ensemble models by accumulating iterations. It uses the cumulative sum of the predicted values of the samples in each tree as the predicted values of the samples in the system. By comparing the predicted results of the model in many research areas, the XGBOOST model has better performance than other models.

The support vector machine [30] is a supervised ML used for binary classification regression and classification subjects. The algorithm is very effective for data with many features. Currently, there are two ways to solve classification problems by carrying out SVM. The first one is to construct several binary classifiers and combine them together. Another is to directly consider the parameter optimization of all classifiers simultaneously. This can effectively avoid the neural network structure and local minima problems and make progress in terms of performance. We set the best parameter by hinge loss function. The equation is shown in (5).

(5) $$L_{hinge}\left(wx+b\right)=max\left(0,1-wx+b\right)$$

The decision tree [31] is a non-parametric supervised learning method for classification and regression. It is a collection of nodes that can make decisions for some feature connected to certain classes. The purpose of the DT is to create a model that predicts the value of a target variable by learning simple decision rules from indicators. DT is also the basis of RF and XGBOOST which is a part of the tree. We built the network by information entropy. The equation is shown in (6).

(6) $$Ent\left(D\right)=-\sum_{k=1}^{2}p_{k}\log_{2}p_{k}$$

In Equation (6), *k* denotes that the number of type and $p_{i}$ means that the percentage of the *i*th type. When the Ent(D) is higher, the purity of *D* is higher.

### 3.6. Setting Parameters of Machine Learning Algorithm

In machine learning models, the parameters need to be selected step by step. GridSearchCV performs a grid search and cross-validation. Grid search means that the parameters are more likely to have better performance than other models by iterating. These parameters are used to train the learner to increase the accuracy of models in the validation set. In this study, we selected several parameters of classification models with reference to papers on machine learning [32,33,34,35,36] as shown in Table 1.

### 3.7. Voting Classifier

**Figure 3:**
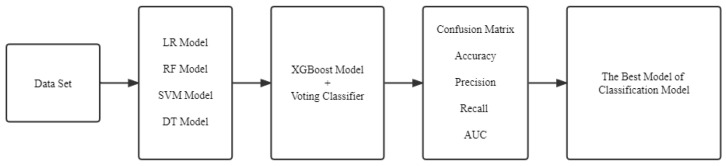
The framework of Voting Classifier.

Voting is an effective and simple ensemble algorithm [37,38]. It is always used in the field of classification when created by combining with at least two or more sub-models. Each classifier model has its own function for testing. Finally, the output was predicted by the mean or the mode of the predictions. Voting classifier included two types, such as hard voting and soft voting. The former one is calculated on the predicted output class. Another one is calculated on the predicted probability of the output class. The framework is as shown in Figure 3.

### 3.8. Performance Metrics

Data sets used in this research contains legitimate and fraudulent records that are labeled as 0 and 1. Therefore, we need to build a confusion matrix and determine several features [39], such as the accuracy (AC), the recall (RC), and the precision (PR). All mathematical functions of these metrics were shown in (7)–(9).

- True Positive (TP): legitimate records that are accurately labeled as legitimate.
- True Negative (TN): fraudulent records that are accurately labeled as fraudulent.
- False Positive (FP): fraudulent records that are incorrectly labeled as legitimate.
- False Negative (FN): legitimate records that are incorrectly classified as fraudulent records.

(7) $$AC=\frac{TP+TN}{TP+TN+FP+FN}$$

(8) $$RC=\frac{TP}{TP+FN}$$

(9) $$PR=\frac{TP}{TP+FP}$$

Apart from that, the Financial Fraud of Listed Company data set is substantially imbalanced. Thus, we need to calculate the Area Under the Curve (AUC) of each model. It is an essential indicator that can measure the performance of fitting of a model. It is called the area under the curve, which refers to a closed area enclosed by the entire ROC curve and the coordinate axis. When the value of AUC the effect in the range of [0.0,0.5), the effect is poor. However, when it is in the range of [0.5,0.7), the effect is better. Finally, when AUC ≈1, the effect is perfect.

## 4. Results and Discussions

### 4.1. Experiment Setup

In this article, ML models are based on Python 3.6. The machine consists of an Intel i7-7700HQ (2.8 GHz) CPU, 16GB RAM, and the Scikit-Learn ML framework.

### 4.2. Results and Discussions

As depicted in Figure 1, we constructed 5 models of feature selection. According to the frequency of each indicators. We selected 13 indicators among all features as shown in Table 2.

According to Table 2, it is obvious that these indicators are related to cash and revenue. In general, these industries need to buy the raw material needed to manufacture for consumers. In that case, pur_fix_assets_oth, cip, advance receipts, noperate_exp, and inventories have a big effect. In the next step, when these products are sold to some buyer, they can obtain the proportion of investment in a short time. Thus, c_paid_to_for_empl are extremely important to the development of listed companies. Finally, when they sold all items and the feedback of consumers is pretty good, the company valuation will increase. Thus, retained_earnings, minority_int, biz_tax_surchfg, assets_disp_gain, basic_eps, compr_inc_attr_m_s, and n_cf_opa_R also have some effect on income of executives, shareholders or investors. We also calculated the point biserial correlation coefficient [40] between these indicators and the dependent variable and the results as shown in Table 3.

We checked the multicollinearity by evaluating with the value of Variance Inflation Factor (VIF). The results showed that these values less than 10. It indicated that there is no multicollinearity among independent variables. Table 3 shows that these indicators have a positive effect on the dependent variable. In addition, the correlation coefficient in the range from 0.4 to 0.9 apart from $x_{12}$. It means that these can have a big impact in predicting and detecting which companies have committed financial fraud.

The training data set contains about 12,642 record items, including 12,526 legitimate records and 116 fraudulent records. As for the testing data set, it contains about 5418 records, including 5354 legitimate records and 64 fraudulent records. After processing by using SMOTE method, the new training data set and testing data set are shown in Table 4.

Before training models, we used five models with imbalanced data sets and balanced data sets, details of results are depicted in Table 4. It is essential to show the importance of the data set process when we use SMOTE in sampling. According to the performance metrics, such as accuracy, recall, precision, and AUC, AUC plays an important role in the fitting of different algorithms. It means that the higher the value, the better the model. Thus, we calculated the AUC when we used the same models with two different data sets. The results are shown in Table 5.

From Table 5, it is clear that the value of AUC in the balanced data set when we applied SMOTE to the original data set is higher than that in the imbalanced data set. In other words, SMOTE is a good way to increase the accuracy when we need to predict and detect companies which have committed financial fraud. At the same time, we reviewed previous works on deep learning [41,42,43]. According to the results of these studies, machine learning has better performance than deep learning in fraud detection. There are three reasons why we did not conduct this research using deep learning.

- Deep learning is more effective in text, video, and image processing compared with classification.
- The importance of each indicator is not clear, because neural networks pay attention to the model framework. It is difficult to prevent and get information of the state of the finances.
- When we conduct deep learning, we cannot know how to predict according to parameters and which indicators are essential for management because it is not easy to explain the connections between neurons.

Thus, we implemented five machine learning algorithms in a balanced data set, such as LR, RF, XGBOOST, SVM, and DT. In this phase, we implemented grid search which can increase the accuracy of prediction and obtain results in a short time. Generally, different models can choose different parameters, as shown in Table 1. The best parameters of each algorithm after training are shown in Table 6.

Based on these results, we used these best models to predict the type of company with training data set. These results are shown in Table 7.

In Table 7, RF is the best model which can accurately predict legitimate company behaviour. In addition, XGBOOST and DT have better performance compared with the other models. However, these models also make some errors in predicting company legitimacy. By contrast, SVM and LR made multiple errors in predicting legitimate or fraudulent company. We also calculated the accuracy, recall, precision, and AUC, to effectively evaluate and choose the best model. These results are shown in Table 8.

**Figure 4:**
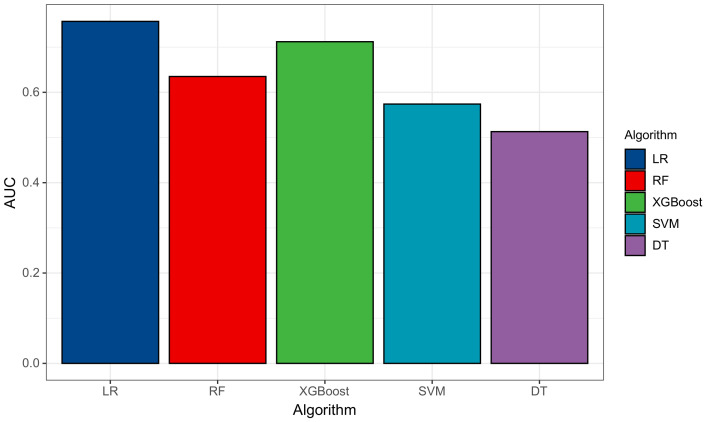
The AUC value of different machine learning algorithms.

In Table 8, RF, XGBOOST, SVM, and DT have better performance in accuracy, recall, and precision compared with LR. In terms of accuracy, all models at over 97%, excluding LR model, for which the accuracy percentage was below 70%. At the same time, the other four models are higher than 97%. In addition, the recall of all models is at about 99%. Then, the precision of these are in the range of from 98% to 100%, excluding the LR model. In conclusion, these models can predict and detect the type of company effectively. However, the AUC value of the five models ranged from 0.50 to around 0.75. We plotted the AUC value as a bar chart to observe and select the best model as the basic model by using Voting in the next step as shown in Figure 4.

As for the AUC of models, when it gets close to 1, it means that the model is pretty good. It is clear that the AUC of logistic and XGBOOST model reached at the highest between 0.7 and around 0.75, which represented an extremely good fitting. However, that of DT is the lowest at around 0.5. In addition, we needed to consider these metrics and decided to chose XGBOOST as the basic model. Then, we combined XGBOOST and Voting with LR, RF, SVM, and DT. We also performed the same procedure as in the first step. The results are shown in Table 9.

In Table 9, it can be seen that all performance metrics made progress in accuracy, recall, precision, and AUC. For accuracy, SVM + XGBOOST is the lowest at 97.933% and that of the other three models are higher than 98%. In addition, the recall for all models is approximately 99%. Thus, the recall of these models is approximately 99%. In other words, the four ensemble models have similar performance, even while there are some differences between many of their performance metrics.

**Figure 5:**
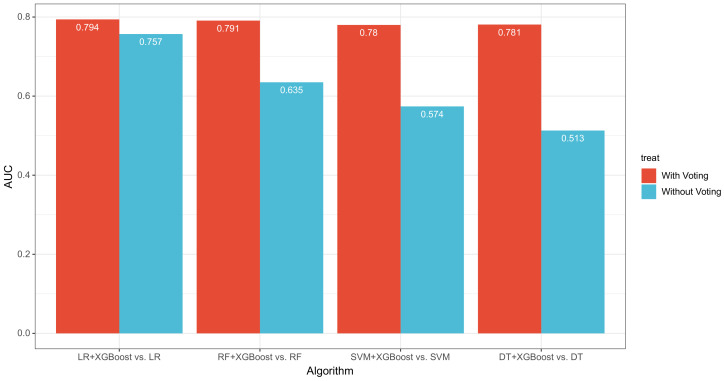
The AUC value of different single machine learning algorithms and ensemble algorithms.

However, it is obvious that the AUC of ML models with Voting made an dramatic improvement and we plotted a bar chart in which this can be clearly observed, shown in Figure 5.

For performance comparison, the AUC of ensemble models is higher than that of a single model, e.g., that of models with Voting at over 0.7. Thus, the maximum AUC difference is 0.268 in between and the average gap is about 0.167. It means that the latter one can predict the class of company accurately and effectively.

**Figure 6:**
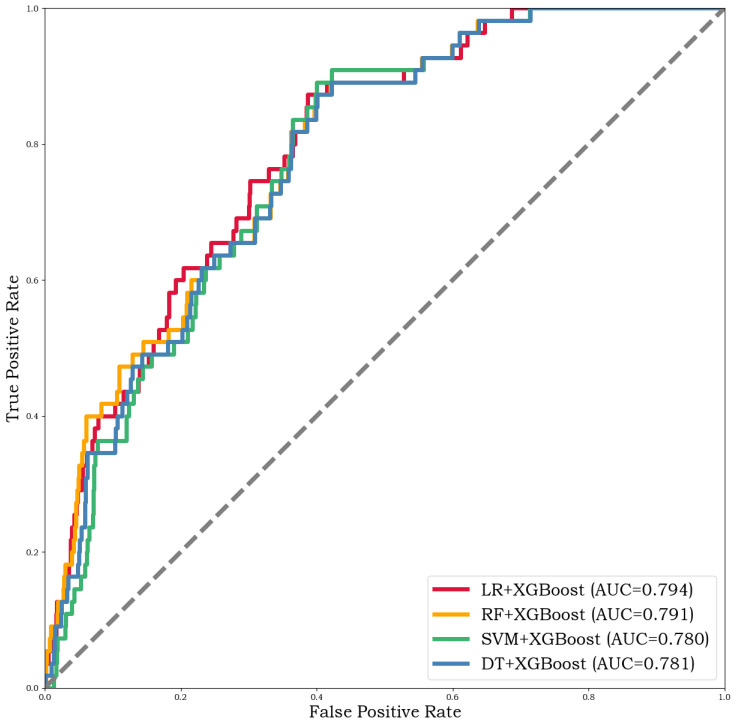
The ROC curve of ensemble algorithms.

Therefore, we used the AUC value as the main metric to choose the optimal model due to the ensemble models performing similarly in terms of accuracy, recall, and precision. We also plotted the ROC curve as shown in Figure 6.

Figure 6 shows that LR+XGBOOST is the best model. As for accuracy, recall, and precision, the values are nearly 100%, respectively. This indicates that the proposed approach is valid and reliable.

Next, a comparison analysis was conducted between the algorithms proposed in the imbalanced data set and previous works, such as k-nearest neighbor (KNN), Multilayer Perceptron (MLP), and Naive Bayes (NB). Other machine learning models built included LR, RF, XGBoost, SVM, and DT. The results are shown in Table 10.

**Figure 7:**
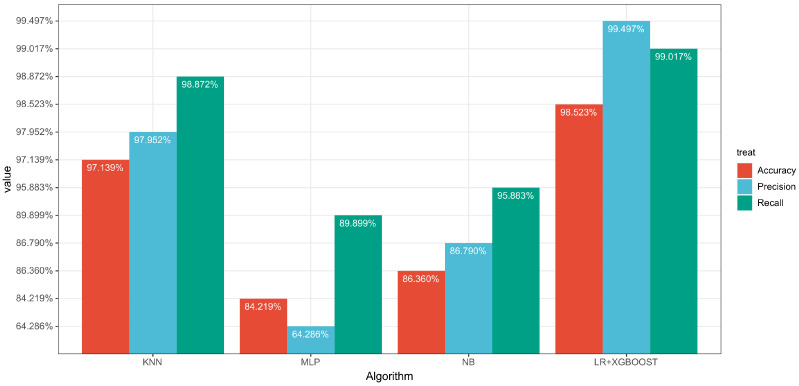
The bar chart of results of existing methods.

In order to illustrate the results, we plotted bar chart as shown in Figure 7.

According to Table 10, the results showed that the accuracy of LR-XGBoost is 14.304% higher than the MLP, 12.163% higher than the KNN, and 1.384% higher than the NB. In terms of recall, LR-XGBoost, KNN, and NB had similar performance when the recall of MLP was at only 89.899%. Finally, there is a big gap in the precision between LR-XGBoost and MLP and NB, 35.211% and 12.707%, respectively. In conclusion, the Voting classifier which combined LR with XGBoost was the best model among those tested.

## 5. Conclusions

This paper proposed an intelligent model to predict and detect the type of company based on the number of samples and various indicators. We carried out wrapper methods for feature selection, including LR, RF, GBDT, DT, and XGBOOST. Next, these indicators were selected by their frequency. In order to classify the type of company, we also built single ML models and ensemble algorithms by using Voting. After building, we chose XGBOOST as basic model and combined others for training. Finally, we evaluated with several performance metrics, such as accuracy, recall, precision, and AUC.

We selected 13 indicators by using machine learning models and counting the frequency of indicators. These indicators were divided into three categories, including the cash flow, operating capacity, and profitability. Cash flow contained six indicators, such as PUR_FIX_ASSETS_OTH, NOPERATE_EXP, C_PAID_TO_FOR_EMPL, INVENTORIES, BIZ_TAX_SURCHFG, and N_CF_OPA_A which are the essential indicators that can evaluate the economic efficiency of projects. During manufacturing or working, managers should pay attention to the outflow and income of cash instead of the size and number of projects. Operating capacity included three indicators, such as CIP, MINORITY_INT, and ASSETS_DISP_GAIN, indicating the ability of the business to increase income by assets. When these indicators are higher, it indicates that the business is operating better. Profitability contained RETAINED_EARNINGS, ADVANCE_RECEIPTS, BASIC_EPS, and COMPR_INC_ATTR_M_S, which indicate the earning capacity. These can reflect not only the operation of the company directly but also whether the capital structure of the company is reasonable. Thus, these have a big impact on different phases of management of a company. At the same time, we set the best parameter of each ML model. The ensemble classifier using Voting showed significant improvement compared with the single model. The best model was the LR+XGBOOST model. The accuracy, recall, and precision being 98.523%, 99.017%, and 99.497%, respectively. Its AUC reached the highest point at 0.794. It means that this ensemble model can predict whether companies have committed financial fraud efficiently and more accurately compared with others.

In conclusion, we designed a scientific method to detect financial fraud and these parameters of machine learning algorithms are reasonable according to the results. This means that this can identify companies which have financial problems with the best accuracy. In addition, it is quite essential for employers working in the finance sector to address doubts regarding financial reports or projects.

At present, the number of listed companies increases year by year due to the rapid development of the economy. Machine learning can greatly reduce the work pressure of staff in identifying whether there are financial fraud problems in listed companies. However, many issues still require deep research deeply as society continues to develop. There are three points we need to follow.

- In terms of collecting data, we ought to collect data as much as possible.
- In term of model application, the financial fraud identification and prediction model can be used not only in the stock market but also in company operations for oversight of the company’s financial status at anytime and anywhere.
- In term of model validation, we plan to collect financial indicators of other listed companies in various industries and apply the model to validate the model effect and ensure its reliability by repeating the experiment.

## Figures and Tables

**Table 1:** The parameter of machine learning models.

Algorithm	Parameter	Value
LR	Penalty	[‘l1’,‘l2’]
	C	[0.01,0.05,0.1,0.5,10,50,100]
	Solver	[‘liblinear’,‘lbfgs’]
RF	Criterion	[‘gini’,‘entropy’]
	Max_features	range(1,len(features))
	N_estimators	[1,10,20,50,100]
XGBOOST	Max_depth	range(1,len(features))
	Learning_rate	[0.01,0.1,0.5,1]
	Gamma	[0.01,0.05,0.1,0.5,10,50,100]
SVM	C	[0.01,0.05,0.1,0.5,10,50,100]
	Gamma	[0.01,0.05,0.1,0.5,10,50,100]
DT	Criterion	[‘gini’,‘entropy’]
	Max_features	range(1,len(features))

**Table 2:** Details of indicators.

Indicator	Meaning	Unit
RETAINED_EARNINGS	Undistributed profits	CNY
PUR_FIX_ASSETS_OTH	Cash paid for fixed assets, intangible assets and other long-term assets	CNY
CIP	Construction work in process	CNY
ADVANCE_RECEIPTS	Deposit received	CNY
NOPERATE_EXP	Non-business expenditure	CNY
C_PAID_TO_FOR_EMPL	Cash received relating to operating activities	CNY
INVENTORIES	Inventory	CNY
MINORITY_INT	Minority equity	CNY
BIZ_TAX_SURCHG	Business taxes and surcharges	CNY
ASSETS_DISP_GAIN	Gain on disposal of assets	CNY
BASIC_EPS	Primary earnings per share	CNY
COMPR_INC_ATTR_M_S	Total comprehensive income attributable to minority shareholders	CNY
N_CF_OPA_R	Operating cash flow (operating income)	CNY

**Table 3:** The pointbiserial correlation coefficient between indicators and dependent variable.

Pointbiserial Correlation Coefficient	$x_{1}$	$x_{2}$	$x_{3}$	$x_{4}$	$x_{5}$	$x_{6}$	$x_{7}$
y	0.908	0.839	0.491	0.665	0.829	0.989	0.552
	$x_{8}$	$x_{9}$	$x_{10}$	$x_{11}$	$x_{12}$	$x_{13}$	
y	0.757	0.432	0.769	0.605	0.116	0.737	

**Table 4:** The number of training and testing data set.

Original	The number of legitimate records	The number of fraudulent records
Training data set	12,526	116
Testing data set	5354	64
**After**	**The number of legitimate records**	**The number of fraudulent records**
Training data set	12,526	12,526
Testing data set	5354	5354

**Table 5:** The performance of AUC in two different data set with same models.

Algorithm	The Value of AUC (Imbalanced Data Set)	The Value of AUC (Balanced Data Set)
LR	0.719	0.757
RF	0.562	0.635
XGBOOST	0.690	0.712
SVM	0.509	0.574
DT	0.505	0.513

**Table 6:** The best parameter of machine learning models.

Algorithm	Parameter	Value
LR	Penalty	l2
	C	50
	Solver	lbfgs
RF	Criterion	gini
	Max_features	1
	N_estimators	50
XGBOOST	Max_depth	7
	Learning_rate	0.5
	Gamma	0.05
SVM	C	100
	Gamma	100
DT	Criterion	gini
	Max_features	9

**Table 7:** Confusion martix of 5 models.

Algorithm	TP	FP	FN	TN
LR	3590	1773	15	40
RF	5362	1	55	0
XGBOOST	5358	5	55	0
SVM	5289	74	55	0
DT	5323	40	54	1

**Table 8:** The results of 5 models.

Algorithm	AC	RC	PR	AUC	Training Time	Testing Time
LR	66.999%	99.584%	66.940%	0.757	0.05 s	0.01 s
RF	98.966%	98.985%	99.981%	0.635	0.26 s	0.01 s
XGBOOST	98.893%	98.984%	99.907%	0.712	1.46 s	0.02 s
SVM	97.619%	98.971%	98.620%	0.574	2.49 s	0.01 s
DT	98.265%	98.996%	99.254%	0.513	0.03 s	0.01 s

**Table 9:** The results of using voting classifier.

Algorithm	AC	RC	PR	AUC	Training Time	Testing Time
LR + XGBOOST	98.523%	99.017%	99.497%	0.794	1.54 s	0.01 s
RF + XGBOOST	98.616%	99.036%	99.571%	0.791	1.75 s	0.05 s
SVM + XGBOOST	97.933%	98.974%	98.937%	0.780	12.34 s	0.71 s
DT + XGBOOST	98.154%	98.995%	99.142%	0.781	1.54 s	0.01 s

**Table 10:** Comparison with existing methods.

Author	Algorithms	AC	RC	PR	Training Time	Testing Time
Kaur et al. [10]	KNN	97.139%	98.872%	97.952%	0.12 s	1.13 s
Kaur et al. [10]	MLP	84.219%	89.899%	64.286%	17.84 s	0.01 s
Kaur et al. [10]	NB	86.360%	95.883%	86.790%	2.38 s	0.52 s
Proposed Method	LR+XGBOOST	98.523%	99.017%	99.497%	1.54 s	0.01 s

## Data Availability

Not Applicable.
